# Effects of Dried Blood Spot Storage on Lipidomic Analysis

**DOI:** 10.3390/molecules23020403

**Published:** 2018-02-13

**Authors:** Cinzia Di Marino, Anna De Marco, Antonio Pisanti, Valeria Romanucci

**Affiliations:** 1 Department of Chemical Sciences, Complesso Universitario di Monte S. Angelo, University of Napoli Federico II, Via Cintia 4, 80126 Napoli, Italy; cdimarin@unina.it; 2 Department of Biology, Complesso Universitario di Monte S. Angelo, University Federico II, Via Cintia 4, 80126 Napoli, Italy; ademarco@unina.it; 3 Consorzio Interuniversitario Sannio Tech, SS Appia km 256, 82030 Apollosa (BN), Italy; antonio.pisanti@tecnobios.com

**Keywords:** dried blood spots, high-throughput, lipidomic, omega-3 biomarker, oxidation

## Abstract

During the lipidomic analysis of red blood cell membranes, the distribution and percentage ratios of the fatty acids are measured. Since fatty acids are the key constituents of cell membranes, by evaluating their quantities it possible to understand the general health of the cells and to obtain health indicators of the whole organism. However, because the analysis is precise, it is necessary to ensure that the blood does not undergo significant variations between the point of collection and analysis. The composition of the blood may vary dramatically weeks after collection, hence, here an attempt is made to stabilize these complex matrixes using antioxidants deposited on the paper cards on which the blood itself is deposited.

## 1. Introduction

A new frontier in the health market is based on the exploration of red blood cell membranes and their lipid composition. Like other “omics”, such as genomics and proteomics, which deal dynamically with molecules that exist in living organisms, lipidomics is the study of not only lipid structures but also the functions and variations that occur during different physiological and pathological conditions [1,2,3,4,5]. Therefore, lipidomics consider lipids as elements that are incorporated into complexes during cellular metabolism and also elements that are related to nutrition.

Lipidomic blood analyses provide a complete and dynamic picture of the cell membrane, this health of the cell membrane is determined by a variety of factors: the lifestyle habits and metabolism of the subject, health conditions, unbalanced diet and cellular stress. At the same time, nutraceutics has been developing in the last few years as a complementary drug business and has been helpful for maintaining physiological conditions, such as the molecular balance of functions with important nutrition factors and preventing cellular degradation, such as that due to stress radicals. However, it is well known that nutraceutical supplementation, dietary supplements, should be regulated as an actual and pharmacological necessity, since the effects of incorrect or excessive intake are similar in both cases. This is especially true for w-3 and w-6 fatty acids. Nutrilipidomics is used to determine fatigue requirements and formulate the most appropriate “cocktail nutraceuticals” to rebalance cell membranes [6,7,8]. Nutrilipidomics is therefore a personalized instrument that, by means of lipidomic analyses, indicates the correct nutritional intervention to restore physiological conditions and to support pharmacological intervention.

There are a number of kits in the market today that enable a user to draw a few drops of blood from a finger and then send the sample, which has been placed on a paper card, to a laboratory for analysis [9,10,11,12]. In addition, globalization has affected the health field, enabling the possibility for analyses to be made thousands of miles away from the patient. Blood, in specific case, travels through normal mail circuits and the time between sampling and sample analysis can take more than 2 weeks. Therefore, it is understood that because the measurement technique is very sensitive, the blood analysis must provide a picture that is as close as possible to the actual conditions. Unfortunately, the matrixes that are analysed are very sensitive to degradation phenomena, primarily that of peroxide oxidation [12,13,14], which can result in huge variations in the determined composition both qualitatively and quantitatively. Fortunately, lipidomic analyses provide a picture of the patient's health by determining not only the individual fatty acids but also parameters such as the saturated fatty acid/monounsaturated fatty acid (SFA/MUFA) ratio, the percent sum of the w-3 fatty acids (Omega 3 Index) [15], the percent sum of the trans fatty acids (Trans Fat Index) [16] and the arachidonic acid/eicosapentaenoic acid (AA/EPA) ratio. In particular, the last cited parameters varied less than the individual fatty acids, guaranteed minimum fluctuations in the time.

As part of our research towards the synthesis, analysis, characterization and use of natural products or their derivatives with high antioxidant power for use in the pharmacology and nutraceutical field [17,18,19,20,21,22,23,24,25,26,27,28], our project aimed to assess the validity of using antioxidants to prevent fatty acid degradation in dried blood spots (DBS). The study is a useful starting point for a detailed assessment of the storage conditions for blood samples prior to their analysis and refutes the use of an antioxidant such as butylated hydroxytoluene (BHT), which has been indicated by other authors [12,29] as being useful for assuring the stability of such complex and sensitive matrixes.

## 2. Results

In this study, the fatty acid composition of the blood, freshly harvested and immediately freeze-dried in the dark, was analysed (3 paper cards for each condition and 2 injections for each paper card) using GC (entry **T0**, Table 1). The **T0** determination served as the baseline for all fifteen storage conditions (entries **A**–**Q**, Table 1).

After fifteen days, samples **A**–**Q** were recovered after extraction from the paper cards, derivatized and then injected into the GC. A parameter set that included the SFA, unsaturated fatty acids (UFA), SFA/UFA, AA/EPA, SFA/MUFA, W-3 index and TFI (Table 2) was obtained from the data reported in Table (see Supplementary Materials). The SFA/UFA ratio is an established factor to consider when evaluating the effect of a 15-day time span on the quali-quantitative composition of blood and blood samples adsorbed on paper cards impregnated with pure or mixed antioxidants. The SFA/UFA ratio of the blood freshly harvested and immediately freeze-dried in the dark (entry **T0**) was 0.62, which was our reference value (Table 3). After fifteen days, the SFA/UFA ratio of the blood adsorbed on the paper and stored in the absence of antioxidants (entry **A**) was 0.68 (Table 3). This increase of 9.7%, was attributed to the oxidation of the unsaturated fatty acids (Table 4).

**Figure 1:**
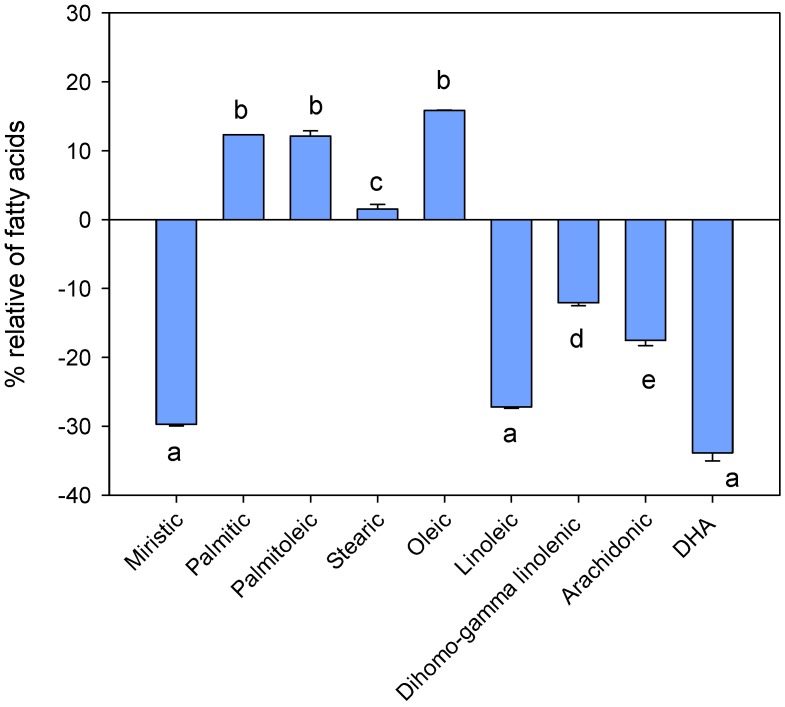
Comparison of the most abundant fatty acids in entry **T0** and **A** (means ± SD, *n* = 6). Different letters indicate statistically significant differences (one-way ANOVA, *p* < 0.05).

Considering only the fatty acids present in fresh blood in quantities greater than 1%, Figure 1 shows that the range varied between −33.9% for the DHA (**22**) and +15.9% for the oleic acid (**7**). Unfortunately, it was not possible to rationalize the results, since the same trends were not even observed for the (positive) saturated and (negative) unsaturated acids, as was expected. The only positive note was that the SFA/UFA ratio increased, as expected, within acceptable limits. For entries **M**, **E**, **C**, **D**, **B** and **I** (Table 1), the SFA/UFA ratio ranged between 0.72 and 0.68, with increases ranging from 9.7 to 16.1% (Table 4).

Interestingly, entries **L**, **N**, **Q, G**, **F**, **P** and **O** exhibited estimated ratios ranging between 0.67 and 0.60, with variations between −3.2 and +8.1% (Table 4). Undoubtedly, we discarded the conditions of entry **H** because the SFA/UFA ratio was equal to 0.54, which was inexplicable, even after taking into account the experimental errors.

Considering the other parameters normally indicated in such analyses, i.e. AA/EPA, W-3 Index, TFI and SFA/MUFA, the variations were remarkable. The variations were very strong, especially in the case of AA/EPA, since EPA was present in very low percentages, less than 1% and even small variations in the quantitative determination resulted in remarkable variations, however, they were not significant. By limiting the discussion to the most interesting (as established for the SFA/UFA ratio) entries, **L**, **N**, **Q**, **G**, **F**, **P** and **O** (Table 1), the following conclusions were deduced: the W-3 index was always underestimated and the decrease in this index ranged from −37% for entry **A** (Table 5) to −7% for entry **N**, the TFI decreased for entries **A**, **Q** and **P** (up to −35%) and increased for the other entries but only entry **N** exhibited a reasonable variation of 6%.

The SFA/MUFA ratio was generally underestimated. Its maximum decrease was −24% but again, entry **N** exhibited the performance, with a decrease of only 9%. The data show that the use of pure antioxidants such as sodium sulphite and ascorbic acid (entries **F** and **G**, respectively) or mixtures such as BHT with ascorbic acid, tartaric acid or sodium sulphite (entries **L**, **N** and **P**, respectively) and sodium sulphite with ascorbic acid or gallic acid (entries **O** and **Q**, respectively) may be beneficial. When individually considered, the 22 fatty acids could undergo significant variations but the parameters considered, which simultaneously accounted for multiple factors, were characterized by oscillations of only a few percentage points, at least for the case of the mixture of BHT and gallic acid (entry **N**). Our data are clearly different from those published in 1979 by Stone et al. which indicated that the use of pure BHT during the routine analysis of fatty acids could ensure stability up to 17 weeks [12].

## 3. Materials and Methods

### 3.1. Materials

All chemicals used were of analytical grade and were purchased from Sigma (St. Louis, MO, USA). The Supelco^®^ standard fatty acid methyl ester (FAME) mixture was purchased from Supelco, Bellefonte, Pennsylvania.

### 3.2. Paper Cards for Blood Collection

Blood was obtained by venepuncture from a single fasting male. Venous blood was collected from the antecubital vein into two 20-mL evacuated tubes (Vacutainer, Becton Dickinson, Franklin Lakes, NJ, USA) by a registered nurse and was then immediately absorbed on a paper card (1.5 cm × 6.0 cm) made of cellulose (Whatman 903, Sanford, ME, USA), such that an area of approximately 1.5 cm^2^ was saturated. The paper cards were pre-soaked in 10 mL solutions containing pure antioxidants or one of six mixture solutions in methanol (entries **B**–**I** and **L**–**Q**, respectively, in Table 1). The solutions were allowed to travel to the top of the paper cards in a thin layer chromatography tank and subsequently removed and dried at room temperature, in the dark, under vacuum over P_2_O_5_. The fatty acid compositions of DBSs were determined using gas chromatography (GC). Then, the paper cards were kept in the same envelopes that are normally sent to analysis laboratories by mail, left at room temperature and analysed after fifteen days.

### 3.3. Fatty Acid Methyl Ester (FAME) Synthesis with MeOH·BF_3_

The DBSs were placed into 16 × 125 mm screw-cap Pyrex culture tubes and 1.0 mL of hexane and 1.0 mL of BF_3_ in MeOH (14%, *wt*/*vol*) were added. The tubes were incubated in a 50 °C water bath for 1 h with vigorous hand-shaking for 10 s every 20 min. Then, 1.0 mL of a saturated solution of NaHCO_3_ and 2.0 mL of hexane were added and the tubes were vortex-mixed. After centrifugation, the hexane layer containing the FAME was placed into a gas chromatography vial that was capped and stored at −20 °C until GC analysis [30,31,32].

### 3.4. Fatty Acid Analysis

The organic layer containing the fatty acid methyl esters was collected, dried, redissolved in a known volume of hexane (100 μL) and analysed using a Shimadzu 2010 series GC-FID (Shimadzu, Milano, Italy). The gas chromatograph was equipped with an SP52-60 capillary column (100 m × 0.25 mm i.d. × 0.20 μm film thickness) with a non-bonded, poly(bis-cyanopropyl siloxane) phase (Sigma Aldrich, St Louis, MO, USA) and nitrogen as the carrier gas. Samples (1.0 μL) were introduced into the injector using an AOC-20i auto sampler (Shimadzu, Milano, Italy) heated to 250 °C with a split ratio of 10:1. The initial temperature was 160 °C with a 2 min hold, followed by a 6 °C/min ramp to 200 °C with a 2 min hold and finally followed by a 6 °C/min ramp to 220 °C with a 25 min hold. The following parameters were set during the experiments: detector temperature: 275 °C, carrier gas: helium for chromatography at a pressure of 1.8 psi, auxiliary gas: hydrogen for chromatography at a pressure of 18 psi, air chromatography at a pressure of 22 psi, and sensitivity of the instrument: 4 to 16 times the minimum attenuation.

### 3.5. Statistical Analysis

Statistical analyses and graphical displays were performed and created, respectively, using SigmaPlot 13.0 software (Jandel Scientific, San Jose, CA, USA). Various fatty acids and fatty acid groups were examined using one-way ANOVA and the significance was inferred for *p* < 0.05. The ANOVA analysis was followed by the Holm-Sidak tests.

## 4. Conclusions

By analysing the lipids of red blood cell membranes, the general health of cells and, therefore, of whole organisms can be understood. The problem is that typically more than 15 days pass between blood collection and analysis as the patient and analytical laboratory can be thousands of miles apart. This manuscript shows that blood preserved without precautions at room temperature undergoes dramatic variations in its composition, providing a distorted picture of the patient's real condition. The effects of antioxidants, pure or in mixtures adsorbed on paper cards on which the blood was deposited, were evaluated 15 days after collection. For all conditions used, the relative % values of 22 fatty acids, the SFA/UFA, AA/EPA and SFA/MUFA ratios and the W-3 Index and Trans Fat Index were determined. The results show that antioxidants are not useful for accurate measurements of individual fatty acids. However, the use of sodium sulphite or ascorbic acid or their mixture with BHT, ascorbic acid or gallic acid, in some cases, enabled the detection previous ratios and indices that were in good agreement with the actual values. Certainly, the use of BHT does not solve the problem, as has been reported in the literature. More studies are needed to determine the best conditions for storing samples of DSBs and at same time, the validity of clinical analysis results that are now routinely performed by public or private laboratories should be questioned.

## Figures and Tables

**Table 1:** Conditions used to store the dry blood spot samples.

Entry	Conditions or Antioxidants Used
**T0**	Sample freshly harvested and immediately freeze-dried in the dark
**A**	Stored in the dark and without antioxidant treatment
**B**	Trolox (3 mg/L, *wt*/*v*)
**C**	Gallic acid (15 mg/L, *wt*/*v*)
**D**	Sodium citrate (15 mg/L, *wt*/*v*)
**E**	Tartaric acid (15 mg/L, *wt*/*v*)
**F**	Sodium sulphite (25 mg/L, *wt*/*v*)
**G**	Ascorbic acid (15 mg/L, *wt*/*v*)
**H**	Butylated hydroxytoluene (BHT) (15 mg/L, *wt*/*v*)
**I**	Orto-phosphoric acid (3 mg/mL, *wt*/*v*)
**L**	BHT/Ascorbic acid 1/1 (*v*/*v*)
**M**	BHT/Gallic acid 1/1 (*v*/*v*)
**N**	BHT/Tartaric acid 1/1 (*v*/*v*)
**O**	Sodium sulphite/Ascorbic acid 1/1 (*v*/*v*)
**P**	BHT/Sodium sulphite 1/1 (*v*/*v*)
**Q**	Sodium sulphite/Gallic acid 1/1 (*v*/*v*)

**Table 2:** Definition of the evaluated parameters.

**SFA** (Saturated Fatty Acids)	Sum of percentage of myristic (**1**), palmitic (**2**), stearic (**5**) and lignoceric (**16**) acids.
**UFA** (Unsaturated Fatty Acids)	Sum of the percentage of trans palmitoleic (**3**), palmitoleic (**4**), trans oleic (**6**), oleic (**7**), trans linoleic (**8**), linoleic (**9**), alpha linolenic (**10**), gamma linolenic (**11**), eicosenoic (**12**), eicosadienoic (**13**), dihomo-gamma linolenic (**14**), arachidonic (**15**), eicosapentaenoic (**17**), nervonic (**18**), docosatetraenoic (**19**), docosapentaenoic (**20**), docosapentaenoic (**21**) and docosahexaenoic (**22**/DHA) acids.
**W-3 Index**	Sum of the percentage of alpha linolenic (**10**), EPA (**17**), docosapentaenoic (**21**) and docosahexaenoic (**22**) acids.
**TFA** (Trans Fatty Acids)	Sum of the percentage of trans oleic (**6**) and trans linoleic (**8**) acids.
**MUFA** (Monounsaturated Fatty Acids)	Sum of the percentage of trans palmitoleic (**3**), palmitoleic (**4**), trans oleic (**6**), oleic (**7**), eicosenoic (**12**) and nervonic (**18**) acids.

**Table 3:** Parameters SFA, UFA, SFA/UFA, AA/EPA, SFA/MUFA, W-3 index and TFI (means ± SD, *n* = 6).

Entry	SFA	UFA	SFA/UFA	AA/EPA	W-3 index	TFI	SFA/MUFA
**T0**	38.23 ± 0.49	61.77 ± 1.78	0.62 ± 0.01	21.02 ± 0.05	2.96 ± 0.11	0.54 ± 0.03	1.34 ± 0.02
**A**	40.64 ± 0.53	59.36 ± 1.63	0.68 ± 0.01	53.21 ± 2.85	1.87 ± 0.05	0.36 ± 0.01	1.26 ± 0.02
**B**	40.81 ± 0.53	59.19 ± 1.71	0.69 ± 0.01	34.82 ± 0.13	2.25 ± 0.06	0.63 ± 0.05	1.26 ± 0.02
**C**	41.14 ± 0.64	58.86 ± 1.65	0.70 ± 0.009	37.61 ± 0.05	2.28 ± 0.07	0.33 ± 0.02	1.34 ± 0.01
**D**	41.02 ± 0.68	58.98 ± 1.98	0.70 ± 0.01	25.07 ± 0.08	2.18 ± 0.07	0.83 ± 0.03	1.25 ± 0.01
**E**	41.59 ± 0.80	58.41 ± 1.70	0.71 ± 0.007	48.12 ± 2.22	2.28 ± 0.08	0.18 ± 0.01	1.25 ± 0.02
**F**	38.72 ± 0.75	61.28 ± 1.76	0.63 ± 0.006	38.07 ± 0.70	2.46 ± 0.08	1.51 ± 0.005	1.23 ± 0.008
**G**	39.58 ± 0.75	60.42 ± 1.71	0.66 ± 0.005	78.68 ± 2.85	1.97 ± 0.05	0.90 ± 0.05	1.12 ± 0.02
**H**	35.25 ± 0.81	64.75 ± 0.91	0.54 ± 0.003	11.16 ± 0.05	4.41 ± 0.09	17.10 ± 0.50	2.23 ± 0.004
**I**	40.64 ± 0.72	59.36 ± 2.07	0.68 ± 0.01	41.83 ± 3.47	2.11 ± 0.12	0.82 ± 0.02	1.28 ± 0.003
**L**	40.09 ± 0.64	59.91 ± 1.86	0.67 ± 0.01	44.18 ± 1.58	2.12 ± 0.07	0.82 ± 0.03	1.26 ± 0.02
**M**	41.80 ± 0.67	58.20 ± 2.03	0.72 ± 0.02	41.40 ± 1.17	2.07 ± 0.06	0.81 ± 0.04	1.32 ± 0.02
**N**	39.60 ± 0.61	60.40 ± 1.66	0.66 ± 0.007	23.00 ± 0.31	2.74 ± 0.07	0.57 ± 0.01	1.22 ± 0.009
**O**	37.36 ± 0.63	62.64 ± 1.67	0.60 ± 0.006	40.69 ± 1.25	2.05 ± 0.09	9.48 ± 0.06	1.02 ± 0.009
**P**	38.31 ± 0.60	61.69 ± 1.64	0.62 ± 0.007	37.78 ± 0.47	2.72 ± 0.06	0.35 ± 0.01	1.25 ± 0.18
**Q**	39.76 ± 0.57	60.24 ± 1.65	0.66 ± 0.009	45.11 ± 1.45	2.27 ± 0.07	0.45 ± 0.02	1.24 ± 0.17

**Table 4:** SFA/UFA values and percentage variations.

Entry	SFA/UFA	Δ%
**T0**	0.62	-
**A**	0.68	+9.7
**M**	0.72	+16.1
**E**	0.71	+14.5
**C**	0.70	+12.9
**D**	0.70	+12.9
**B**	0.69	+11.3
**I**	0.68	+9.7
**L**	0.67	+8.1
**N**	0.66	+6.5
**Q**	0.66	+6.5
**G**	0.66	+6.5
**F**	0.63	+1.6
**P**	0.62	+0.0
**O**	0.60	−3.2
**H**	0.54	−12.9

**Table 5:** Different parameters in the best conditions to storage the DBS.

Entry	AA/EPA	W-3 Index	TFI	SFA/MUFA
**T0**	21.02	2.96	0.54	1.34
**A**	53.21 (153%)	1.87 (−37%)	0.36 (−33%)	1.26 (−6%)
**L**	44.18 (44%)	2.12 (−28%)	0.82 (52%)	1.26 (−6%)
**N**	23.00 (9%)	2.74 (−7%)	0.57 (6%)	1.22 (−9%)
**Q**	45.11 (77%)	2.27 (−23%)	0.45 (−17%)	1.24 (−7%)
**G**	78.68 (128%)	1.97 (−33%)	0.90 (67%)	1.12 (−16%)
**F**	38.07 (22%)	2.46 (−17%)	1.51 (180%)	1.23 (−8%)
**P**	37.78 (44%)	2.72 (−8%)	0.35 (−35%)	1.25 (−7%)
**O**	40.69 (52%)	2.05 (−31%)	9.48 (>>100%)	1.02 (−24%)

In parentheses the % difference from the **T0**, considered the reference value.

## Supplementary Materials

The supplementary materials are available online. Table S1: Relative amount (%) of fatty acids (**1**–**22**) by GC analysis.
