# Supplementary material for: Effects of Dried Blood Spot Storage on Lipidomic Analysis
Source: Molecules. 2018 Feb 13;23(2):403. doi: 10.3390/molecules23020403 (PMC6017148; doi:10.3390/molecules23020403)
Supplement: Supplementary file 1 [file molecules-23-00403-s001.pdf]

Table S1. Relative amount (%) of fatty acids (**1–22**) by GC analysis (means ± SD; *n* = 6).

	**Fatty acid**	**T0**	**A**	**B**	**C**	**D**	**E**	**F**	**G**	**H**	**I**	**L**	**M**	**N**	**O**	**P**	**Q**
**1**	Miristic (14:0)	1.101 ± 0.051	0.774 ± 0.033	0.889 ± 0.041	0.992 ± 0.065	1.083 ± 0.089	1.024 ± 0.100	0.847 ± 0.088	1.447 ± 0.066	0.443 ± 0.045	1.481 ± 0.032	0.845 ± 0.044	1.278 ± 0.077	0.732 ± 0.031	0.876 ± 0.098	0.967 ± 0.099	1.020 ± 0.053
**2**	Palmitic (16:0)	26.940 ± 0.123	30.258 ± 0.133	29.430 ± 0.142	30.093 ± 0.201	29.509 ± 0.232	29.594 ± 0.321	27.130 ± 0.234	20.956 ± 0.213	26.098 ± 0.289	28.820 ± 0.202	28.886 ± 0.199	29.460 ± 0.197	28.647 ± 0.301	27.474 ± 0.167	28.110 ± 0.161	28.939 ± 0.200
**3**	Trans palmitoleic (16:1n7t)	0.288 ± 0.016	0.304 ± 0.021	0.362 ± 0.022	0.329 ± 0.031	0.355 ± 0.029	0.260 ± 0.031	0.339 ± 0.022	1.133 ± 0.028	0.449 ± 0.031	0.710 ± 0.026	0.711 ± 0.031	0.703 ± 0.032	0.351 ± 0.012	0.295 ± 0.028	0.770 ± 0.015	0.695 ± 0.022
**4**	Palmitoleic (16:1n7)	2.607 ± 0.089	2.924 ± 0.122	3.160 ± 0.100	2.893 ± 0.144	2.362 ± 0.213	4.322 ± 0.111	2.534 ± 0.189	4.920 ± 0.121	12.119 ± 0.146	3.046 ± 0.333	3.053 ± 0.178	3.018 ± 0.154	3.822 ± 0.201	2.673 ± 0.199	3.178 ± 0.123	2.662 ± 0.100
**5**	Stearic (18:0)	9.339 ± 0.345	9.484 ± 0.421	10.314 ± 0.399	9.829 ± 0.444	10.184 ± 0.429	10.828 ± 0.456	10.466 ± 0.510	16.924 ± 0.611	8.422 ± 0.457	10.145 ± 0.467	10.168 ± 0.500	10.873 ± 0.399	10.234 ± 0.419	8.817 ± 0.389	9.085 ± 0.412	9.717 ± 0.378
**6**	Trans oleic (18:1t)	0.420 ± 0.021	0.1250.006	0.224 ± 0.040	0.117 ± 0.004	0.201 ± 0.003	0.111 ± 0.004	0.565 ± 0.002	0.494 ± 0.014	0.054 ± 0.016	0.178 ± 0.002	0.179 ± 0.002	0.177 ± 0.003	0.223 ± 0.003	9.296 ± 0.001	0.178 ± 0.009	0.217 ± 0.008
**7**	Oleic (18:1n9)	24.640 ± 0.789	28.547 ± 0.897	28.184 ± 0.880	26.827 ± 0.678	29.330 ± 0.899	28.358 ± 0.788	27.405 ± 0.878	28.014 ± 0.754	0.224 ± 0.079	27.479 ± 0.006	27.542 ± 0.911	27.231 ± 0.855	27.594 ± 0.666	24.065 ± 0.776	26.038 ± 0.883	28.040 ± 0.854
**8**	Trans linoleic (18:2n6t)	0.118 ± 0.008	0.238 ± 0.009	0.407 ± 0.011	0.212 ± 0.016	0.630 ± 0.007	0.065 ± 0.008	0.942 ± 0.004	0.404 ± 0.041	17.043 ± 0.005	0.640 ± 0.561	0.642 ± 0.034	0.635 ± 0.045	0.347 ± 0.011	0.191 ± 0.006	0.173 ± 0.007	0.231 ± 0.011
**9**	Linoleic (18:2n6)	18.964 ± 0.567	13.810 ± 0.456	14.843 ± 0.459	15.666 ± 0.500	14.513 ± 0.654	13.599 ± 0.546	14.026 ± 0.455	11.824 ± 0.433	9.600 ± 0.355	13.551 ± 0.467	13.582 ± 0.489	12.898 ± 0.767	14.698 ± 0.653	14.053 ± 0.348	16.407 ± 0.345	15.039 ± 0.435
**10**	Alpha linolenic (18:3n3)	0.230 ± 0.009	0.018 ± 0.008	0.416 ± 0.002	0.375 ± 0.005	0.422 ± 0.004	0.297 ± 0.012	0.188 ± 0.011	0.183 ± 0.002	1.489 ± 0.012	0.061 ± 0.008	0.061 ± 0.001	0.041 ± 0.002	0.530 ± 0.012	0.336 ± 0.023	0.463 ± 0.011	0.079 ± 0.009
**11**	Gamma linolenic (18:3n6)	0.076 ± 0.006	0.351 ± 0.009	0.164 ± 0.011	0.212 ± 0.008	0.192 ± 0.005	0.132 ± 0.009	0.292 ± 0.011	0.413 ± 0.009	1.072 ± 0.012	0.155 ± 0.056	0.156 ± 0.005	0.154 ± 0.009	0.357 ± 0.011	0.191 ± 0.012	0.208 ± 0.014	0.204 ± 0.011
**12**	Eicosenoic (20:1n9)	0.079 ± 0.005	0.304 ± 0.011	0.195 ± 0.012	0.238 ± 0.009	0.317 ± 0.021	0.153 ± 0.022	0.366 ± 0.011	0.487 ± 0.014	2.342 ± 0.011	0.304 ± 0.089	0.305 ± 0.011	0.301 ± 0.010	0.308 ± 0.009	0.214 ± 0.012	0.421 ± 0.011	0.413 ± 0.013
**13**	Eicosadienoic (20:2n6)	0.628 ± 0.034	2.145 ± 0.033	0.275 ± 0.010	0.802 ± 0.090	0.369 ± 0.023	0.089 ± 0.011	2.062 ± 0.005	1.691 ± 0.078	8.101 ± 0.012	1.544 ± 0.321	1.547 ± 0.077	1.530 ± 0.056	0.880 ± 0.034	0.742 ± 0.066	1.791 ± 0.087	0.589 ± 0.031
**14**	Dihomo-gamma linolenic (20:3n6)	1.416 ± 0.034	1.245 ± 0.023	1.081 ± 0.034	1.204 ± 0.021	1.199 ± 0.022	1.130 ± 0.024	1.234 ± 0.022	0.880 ± 0.024	0.988 ± 0.019	1.175 ± 0.028	1.178 ± 0.026	1.165 ± 0.029	1.338 ± 0.031	1.137 ± 0.055	1.311 ± 0.034	1.316 ± 0.045
**15**	Arachidonic (20:4n6)	8.027 ± 0.231	6.619 ± 0.123	6.892 ± 0.202	6.962 ± 0.199	5.968 ± 0.204	6.617 ± 0.187	7.553 ± 0.188	7.050 ± 0.222	6.484 ± 0.156	7.312 ± 0.211	7.741 ± 0.178	7.171 ± 0.189	7.182 ± 0.121	6.757 ± 0.200	7.273 ± 0.177	7.416 ± 0.188
**16**	Lignoceric (24:0)	0.798 ± 0.033	0.125 ± 0.002	0.173 ± 0.006	0.226 ± 0.009	0.241 ± 0.011	0.143 ± 0.012	0.275 ± 0.009	0.251 ± 0.011	0.291 ± 0.009	0.193 ± 0.012	0.194 ± 0.011	0.191 ± 0.009	0.119 ± 0.007	0.202 ± 0.050	0.144 ± 0.003	0.081 ± 0.004
**17**	Eicosapentaenoic/ EPA (20:5n3)	0.382 ± 0.012	0.124 ± 0.010	0.198 ± 0.005	0.185 ± 0.005	0.238 ± 0.009	0.138 ± 0.011	0.198 ± 0.009	0.090 ± 0.009	0.581 ± 0.013	0.175 ± 0.034	0.175 ± 0.011	0.173 ± 0.010	0.312 ± 0.010	0.138 ± 0.008	0.193 ± 0.002	0.164 ± 0.010
**18**	Nervonic (24:1n9)	0.488 ± 0.033	0.024 ± 0.001	0.239 ± 0.002	0.191 ± 0.009	0.127 ± 0.007	0.010 ± 0.001	0.188 ± 0.009	0.171 ± 0.012	0.604 ± 0.011	0.130 ± 0.023	0.130 ± 0.005	0.129 ± 0.008	0.227 ± 0.010	0.171 ± 0.003	0.092 ± 0.005	0.062 ± 0.003
**19**	Docosatetraenoic (22:4n6)	0.791 ± 0.034	0.727 ± 0.033	0.723 ± 0.042	0.694 ± 0.043	0.595 ± 0.029	0.789 ± 0.034	1.045 ± 0.045	0.800 ± 0.043	0.611 ± 0.044	0.840 ± 0.039	0.842 ± 0.045	0.833 ± 0.038	0.004 ± 0.001	0.623 ± 0.035	0.783 ± 0.044	0.732 ± 0.037
**20**	Docosapentaenoic (22:5n6)	0.269 ± 0.009	0.125 ± 0.023	0.192 ± 0.011	0.226 ± 0.012	0.645 ± 0.021	0.501 ± 0.034	0.272 ± 0.031	0.170 ± 0.022	0.645 ± 0.021	0.183 ± 0.022	0.184 ± 0.009	0.182 ± 0.008	0.191 ± 0.010	0.202 ± 0.023	0.352 ± 0.009	0.354 ± 0.012
**21**	Docosapentaenoic (22:5n3)	0.458 ± 0.031	0.476 ± 0.022	0.538 ± 0.021	0.623 ± 0.023	0.412 ± 0.031	0.533 ± 0.024	0.743 ± 0.022	0.492 ± 0.034	1.460 ± 0.023	0.571 ± 0.056	0.572 ± 0.019	0.566 ± 0.021	0.587 ± 0.017	0.558 ± 0.018	0.571 ± 0.0 11	0.642 ± 0.013
**22**	Docosahexaenoic/ DHA (22:6n3)	1.892 ± 0.066	1.251 ± 0.019	1.101 ± 0.044	1.103 ± 0.045	1.108 ± 0.036	1.310 ± 0.039	1.329 ± 0.051	1.204 ± 0.047	0.878 ± 0.053	1.304 ± 0.034	1.307 ± 0.045	1.293 ± 0.034	1.315 ± 0.043	0.989 ± 0.055	1.492 ± 0.041	1.389 ± 0.045
